# Shifting gears higher - digital slides in graduate education - 4 years experience at Semmelweis University

**DOI:** 10.1186/1746-1596-5-73

**Published:** 2010-11-22

**Authors:** László Fónyad, László Gerely, Mária Cserneky, Béla Molnár, András Matolcsy

**Affiliations:** 1 1st. Dept. of Pathology and Experimental Cancer Research, Semmelweis University, Üllői st. 26., H-1085 Budapest, Hungary; 2 3DHISTECH Ltd., Konkoly-Thege Miklós road 29-33., Building 18. H-1121 Budapest, Hungary; 3 Second Department of Internal Medicine, Semmelweis University, Üllői st. 26., H-1085 Budapest, Hungary

## Abstract

**Background:**

The spreading of whole slide imaging or digital slide systems in pathology as an innovative technique seems to be unstoppable. Successful introduction of digital slides in education has played a crucial role to reach this level of acceptance. Practically speaking there is no university institute where digital materials are not built into pathology education. At the 1st. Department of Pathology and Experimental Cancer Research, Semmelweis University optical microscopes have been replaced and for four years only digital slides have been used in education. The aim of this paper is to summarize our experiences gathered with the installation of a fully digitized histology lab for graduate education.

**Methods:**

We have installed a digital histology lab with 40 PCs, two slide servers - one for internal use and one with external internet access. We have digitized hundreds of slides and after 4 years we use a set of 126 slides during the pathology course. A Student satisfaction questionnaire and a Tutor satisfaction questionnaire have been designed, both to be completed voluntarily to have feed back from the users. The page load statistics of the external slide server were evaluated.

**Results:**

The digital histology lab served ~900 students and ~1600 hours of histology practice. The questionnaires revealed high satisfaction with digital slides. The results also emphasize the importance of the tutors' attitude towards digital microscopy as a factor influencing the students' satisfaction. The constantly growing number of page downloads from the external server confirms this satisfaction and the acceptance of digital slides.

**Conclusions:**

We are confident, and have showed as well, that digital slides have got numerous advantages over optical slides and are more suitable in education.

## Background

Pathology is a discipline dealing with the development of diseases, their aetiology and effects on the human body, being closely correlated to prevention, diagnosis and therapy. It is one of the most important medical disciplines, connecting morphological changes to clinical aspects, therefore the study and knowledge of pathology is essential for the understanding of clinical subjects. Pathology courses - and teaching in general - is facing many challenges today, and has to deal with several intriguing questions, like:

1. How could the different pathology curriculums (graduate and postgraduate) set by the governments, integrate the constantly growing knowledge of sciences with precise standards while preserving the lucidity and manageability of the topics the students have to master?

2. How could universities apply different teaching and learning methods (For example: problem based learning, inquiry-based learning, augmented learning, cooperative learning, distance learning, e-learnig etc.)?

3. How could the advances of computer sciences be maximized in pathological education?

Answering the first two questions is far beyond the prospect of this paper. For the third question the spreading of digital microscopes in all fields of pathology [1-8] especially in the histopathology classrooms Worldwide may provide a suitable answer [9-12].

### Pathology curriculum framework for graduate education in the medical course of the 1st. Department of Pathology and Experimental Cancer Research, Semmelweis University

We have designed a pathology curriculum that fundamentally has a clinico-pathological point of view. During our lectures, seminars and practices we aim to provide an approach that will enable our students to develop a medical way of thinking, to formulate evidence based judgements, and enhance their diagnostic skills. Pathology at Semmelweis University is a 2 semester subject covering 224 teaching hours for lectures and practices.

#### Lectures

Two 90 minute long lectures are held each week. The topics discussed in the first semester cover basic pathological changes, and the second semester covers the diseases of the different organ systems (detailed organ specific pathology).

#### Practices

There are similarly two 90 minute long practices weekly. The practical training consists of 30 autopsy practices, 18 histology seminars and 8 specimen consultations. The goal of the autopsy practices is to describe the basic pathomorphological changes based on the previous anatomic knowledge, and to correlate the morphological findings with the clinical picture. After the autopsy is performed each case is discussed in situ with the clinicians. Students are taking an active part in such consultations. The aim of histological practices is to describe and recognize the fundamental histopathological alterations. For specimen consultation we have formalin fixed and re-colourised autopsy specimens using *Romhanyi's *technique, demonstrating the rare and interesting pathological entities that students do not necessarily meet with during routine autopsies.

#### Type of examination

Both semesters are concluded with practical (autopsy, histology and specimen recognition) and oral (two topics and explanation of one autopsy report) examination. Written tests are not used for examination.

#### Digital solutions in the practices

In the autopsy room we use interactive smart tables, that are connected to the laboratory information system of the University. All the clinical data of the patients (e.g. X-ray, CT, MRI images along with videos of angiography and different scope techniques) can be displayed. For histology practices we use digital slides.

The aim of the paper is to summarize our experiences gathered with the installation of a fully digitized histology lab for graduate education.

## Methods

### Key hardwares and softwares used

**Figure 1:**
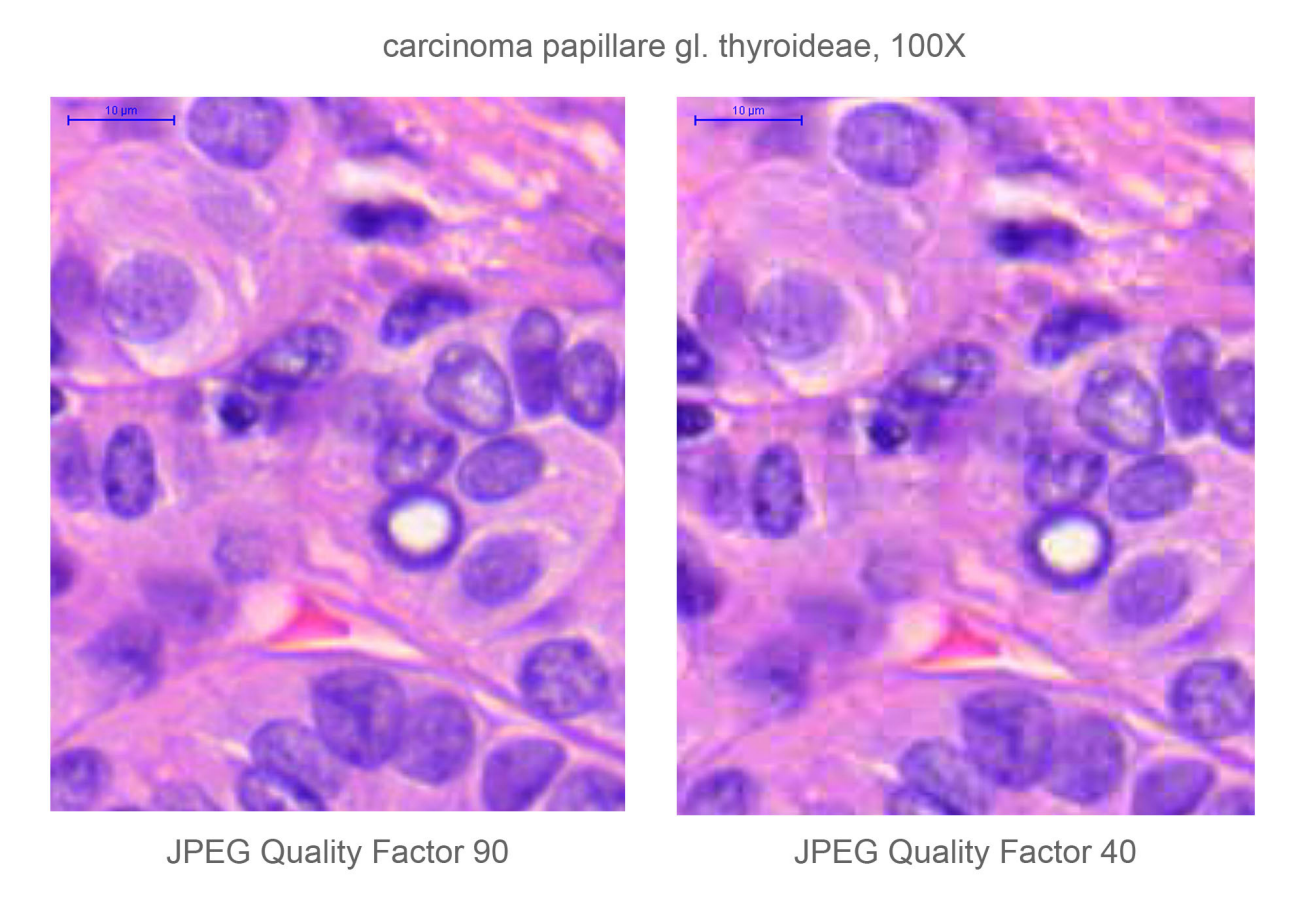
**Difference between the resolution of the original (JPEG Quality factor compression 90) and the compressed (JPEG Quality factor compression  40) digital slide, depicting the same region of a papillary thyroid cancer. (HE-stain, 100× magnification.)** The pixelation is clearly visible on the right image, after compressing the digital slide.

After a testing period lasted for almost a year, including software tests and pilot histology practices, we decided to replace all optical microscopes by computers for the 2007 academic year. We set up a digital histology lab with 40 commercially available PCs (Intel 3.06 GHz processor, 1 GB DDRII RAM, TFT 17" LCD monitor), an internal slide server (AMD Phenom 9550 Quad Core 2.2 GHz processor, 2 GB FBD DDR2 RAM) and built up the intranet (Cisco 2970G 24TS-E switch, 1000 Mbps) that connects the PCs with the teacher's laptop. Mirax Viewer on the client PCs and Mirax Slide Server were installed on. We collected, revised and digitised ~1000 slides from our archive, representing typical histological alterations, and selected the best ones to be used. The slide set contained mainly H&E slides but the material was completed with special stains and immunohistochemistry (IHC) slides as well. In case of necessity re-cut and re-stain were performed. Additionally with the aid of recognised members of the Hungarian Society of Pathologist representative slides from various pathology departments of Hungary we also received. The configuration of the scanner has been changing continuously as new developments reveal, but generally the slides were scanned using MIRAX Scan equipped with a Hitachi 3-chip camera and a Plan-Apochromat objective with 20× magnification, 0.465 μm/pixel resolution, 0.63 numeric aperture, 0.5× camera adapter magnification and 1× optovar magnification. The slide format was *.mrxs, set for 90 JPEG Quality factor. After the scanning process the digital slides were modified to perfectly fit for the needs of the digital histology lab and the intranet. Tile size was resized to 1024 × 1024 pixels and various compressions were performed in order to reduce file size to maintain acceptable speed for the consultation. The compression left the file format, reducing the JPEG Quality factor from 90 to 40. (Figure 1) We have a compilation of 126 slides for the students that are discussed during the 18 histology seminars of the 2 semesters of pathology training.

### Typical scenario of a histology class using digital slides

**Figure 2:**
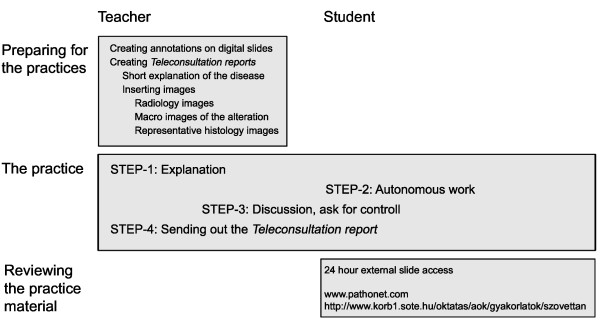
**Scheme of the teaching - learning assessment**.

At the beginning of the practice the students log into the internal slide server and connect to a teleconsultation group previously created by the teacher via the intranet. During histology classes each disease is discussed in 4 steps. (Figure 2) First the basic clinico-pathological aspects are explained then the teacher opens the slide(s) and share it(them) with the students. Browsing over the slide is controlled by the teacher and the same region on the same magnification appears on the client computers as well as with an additional prominent cursor representing the location of the teacher's cursor on the slide. The students could only look around on the actual screen using their cursor to highlight the part where they are pointing in a small magnifier window. After this guided part the students disable leader control and review the slide individually. When finding a structure or an alteration not fully understood, the students are able to request control over the consultation. After the teacher granted the control for a period of time, a student becomes a leader of the whole consultation opening the door for the teacher to explain the problematic part to everybody in the classroom. The control could always be taken back by the teacher. It is possible to annotate areas on the slides and add short texts and comments for them. Annotations could be placed before the practices or when they are made during the practices they appear on all slides simultaneously. A chat box in the software allows the teacher for spelling difficult medical terms. Students can take screenshots of the slides using a built in image capture function and save them on a pen drive. At the end of the class there is a possibility to create a report of the consultation. In this *.rtf document, thumbnail views of the slides and views of selected annotated areas appear with comments. Such documents could be prepared in advance of the practices and imported for the consultation. In this way professionally edited reports could be prepared completing them with tables, radiology images, macro images etc.

### External access for the slides

For security reasons the internal server supplying the practices can not yet be reached from outside the institute. To provide the students with continuous external access service to the educational material the slides were uploaded to http://www.pathonet.com an open access web portal providing free storage capacity for digital slides. The portal runs on 2 separate server computers, one for storing the web-pages and all text data and one for storing and handling the digital slides. The first is an Intel Xeon 2 GHz, 1 GB RAM server running on Linux, the second one is a dual Intel XEON 5150, 2 GB RAM, 4 TB HDD, running on Win2003. The slides can be opened with a free version of Mirax Viewer or with a simple Java Viewer. In the first year a handout for the slides were uploaded to the server too. Later these materials were deleted because of the educational policy of the Institute.

### Histology examinations using digital slides

For the midterm and final examinations we use digital slides. A dedicated software - E-School Exam - was installed on the server. The software allows editing and compiling question sets for test papers containing close ended (simple choice question, multiple choice question) and open ended questions as well. Both the questions and the answers appear in a random order for every user after authentication, login to the server and starting an examination. Digital slides can also be linked to questions. The answer for such a question could be an image captured and uploaded by the student. From the numerous possibilities the software offers, we use only its slide linking and randomising capability.

### Feed backing approaches

**Figure 3:**
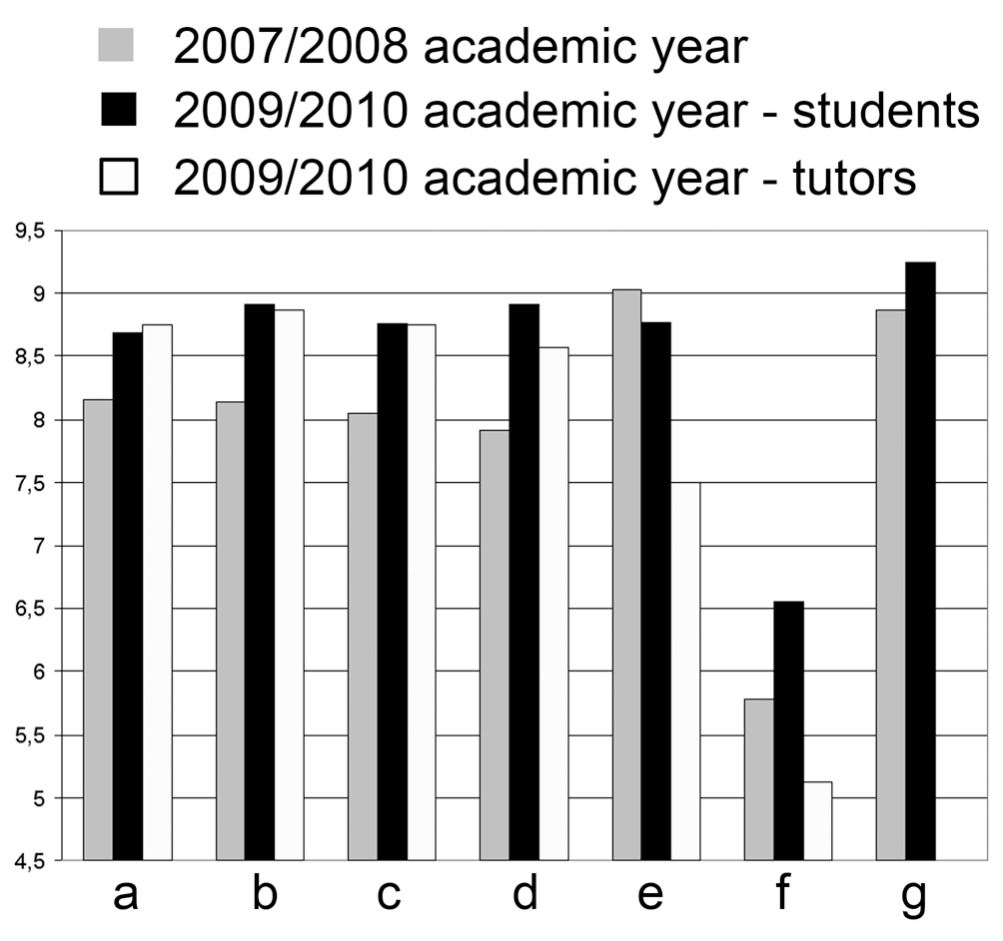
**Results from the Student/Tutor satisfaction questionnaire**. Answers for all questions were scaled between 1-10, where the higher score preferred digital over conventional microscopy and 5 denoted no difference between the methods. a- How much have you liked the practices with digital slides? b- Comparing to a conventional microscope, how much have you liked working with digital slides? c- Have you found the application user-friendly? d- Comparing to a conventional microscope, how user-friendly the application was? e- How do you rate the quality of the digital slides? f- How do you rate the speed of opening a slide during practices? g- How useful the Pathonet was for preparing the examination? (1-10: not useful - very useful).

**Figure 4:**
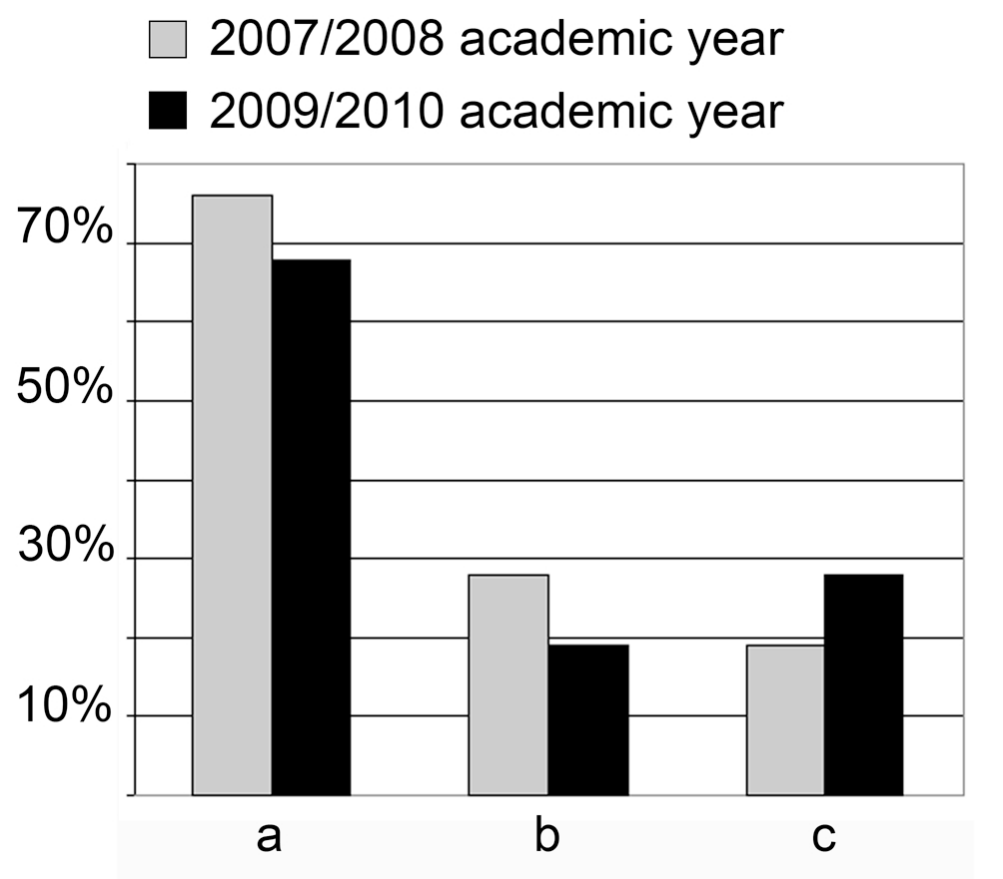
**Student activity index**. a- Are you disconnecting the consultation in order to browse the slide for yourself? b- Are you requesting the control of the consultation? c- Are you taking pictures from the slides for your own?

We have composed a Student satisfaction questionnaire and a Tutor satisfaction questionnaire, both to be completed voluntarily. (See the questions in Figure 3, and Figure 4.) The main interest was to disclose the students'/teachers' opinion about the user friendliness of the software used, the image quality of digital slides and how they make use of the many features digital slides offer. During the first and second year the students attend anatomy courses where only optical microscopes are used for teaching purposes enabling them to compare the optical and digital microscopy techniques. We counted an overall satisfaction rate, by adding the results of every question from the questionnaire and correlated it to a maximum possible score, naming this rate "attitude". We measured this attitude towards digital applications in education for tutors and compared this result with the attitude of the tutors' students when at least 10 students of the specific tutor filled the questionnaire. The website attendance of the http://www.pathonet.com portal was also measured. We have collected data after the first and the third year of integration digital slides into education.

## Results

In the past 3 years we had 928 students registered for pathology courses including students of the Hungarian and English medical and Hungarian and English dental program. During this period the digital histology classroom served approximately 1600 hours.

### Student/tutor satisfaction questionnaire

**Figure 5:**
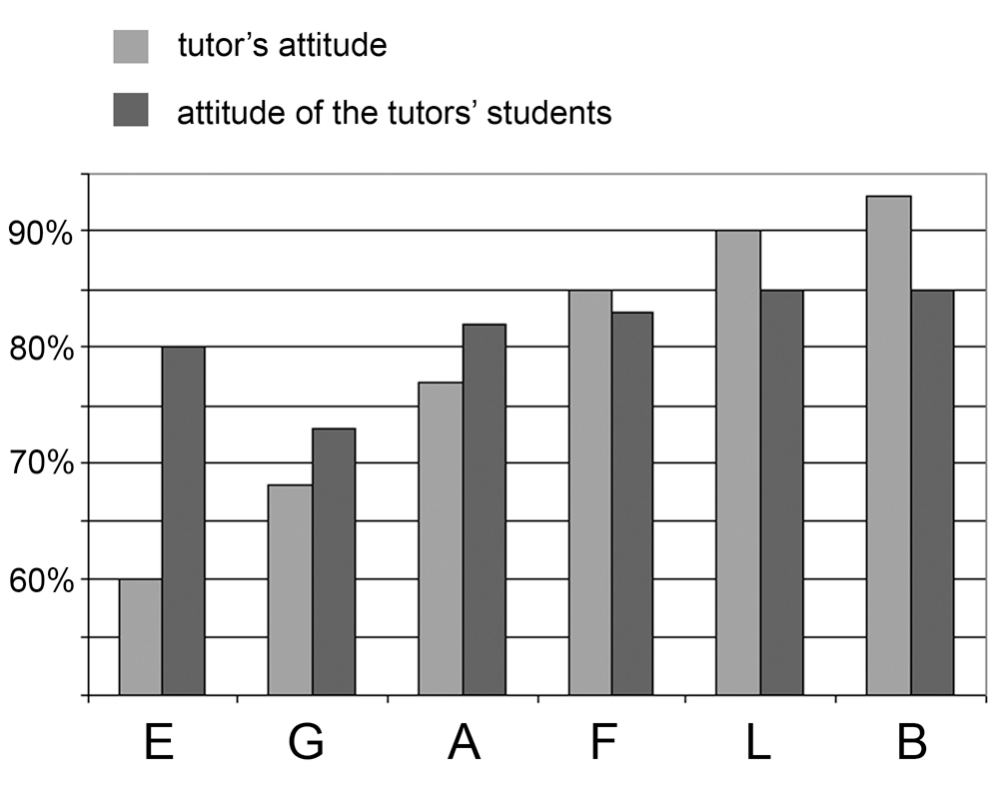
**Tutors' and students' attitude towards digital applications in education**. Capitals (E, G, A, F, L, B) represent the 6 tutors where more than 10 student filled the questionnaire. Responding students n = 16(E), 12(G), 18(A), 16(F), 11(L), 13(B).

After the first year 116/268 students, after the third year 112/334 students and 8/12 tutors filled the questionnaires. The results are detailed in Figure 3, and show a great satisfaction with digital slides. The results show the consequence of the above mentioned file compression (Figure 1) effecting slide quality (decrease) and the speed of opening slide (increase). Tutors were more critical of the quality and the speed than the students. Unfortunately less students are taking part actively in the practices (disconnecting or requesting control), but more are using the image capture function of the software. (Figure 4) There was a correlation between the teachers' and the students' attitude towards digital applications. The more the teachers were confident the better student attitude was recorded. (Figure 5)

### Page load statistics of the Pathonet portal

In the queries there were some questions related to the usage of the Pathonet portal. According to the students' answers in 2007/2008 97% of the students used the site and found it very useful to prepare for the examinations (8.86/10). The query this year ended similar results, respectively 98% and 9.22/10.

**Figure 6:**
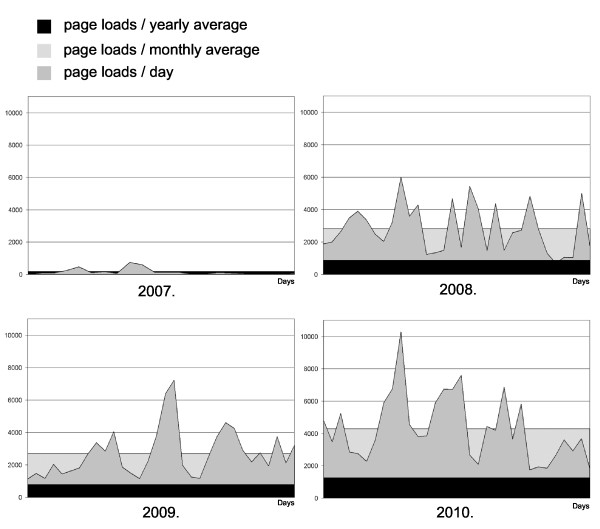
**Page loads (n) from Pathonet (2007-2008-2009-2010 summer - same 30 days period)**. Number of students in the pathology courses: 2007/2008 = 268, 2008/2009 = 326, 2009/2010 = 334.

The increase in the number of page loads from the portal supports the results of the somehow subjective results of the queries. Figure 6 shows page loads from the same periods of the years 2007-2008-2009-2010. Peaks represent typical attributes before examination-days.

## Discussion

The success of digital microscope applications have brought a new era to medicine, especially to anatomic pathology, both in the field of research, education and routine practice. Considering the fact that the most obvious advantages of such systems appear in education it is not surprising that spreading of digital microscopes have started in the histopathology classrooms [13]. Our results additionally show that digital slides could easily be integrated to histopathology education. The results emphasize the importance of the "human factor" in the efficiency of the installation of any new techniques confirmed by how the tutors' attitude towards digital slides affected the students' attitude. (Figure 5) Without overestimating ones age on the impact on the capability to accept and apply new innovations we have to mention that the younger the tutor was the better results were detected and vice versa. Even though the image compression resulted in a slight corruption of image quality (and an increase in the speed of slide handling) we think it is a reasonable compromise as this decline only appears in such high magnifications which a regular optical microscope in a classroom not even reaches equipped with a 20× or maximum 40× objective. The constantly increasing number of page loads from our slide server also confirms our faith in putting extra efforts to manage the portal.

In order to understand this success in pathology education we have to inspect the advantages of digital microscopes and slides over optical microscopes and slides and the possible disadvantages. Some may argue that necessity of additional investments in education (to equip a room with computers, build up a network, buy a slide scanner) is a disadvantage. Furthermore as long as digital microscopes does not replace optical ones in routine practice it could happen that a pathology resident would use a light microscope for the first time, the first day of their pathology residencies [13]. We are convinced that the advantages of digital slides in education far outweigh the mentioned possible drawbacks. What are these advantages? Some of them could be defined as features that glass slides and optical microscopes bear as well, but with digital slides the quality of those features is increased. The next group of attributes is unique to digital slides, providing a powerful tool to teachers that is impossible to be managed using optical microscopy.

Features of digital slides providing higher quality in education or allowing simplification of the processes:

### Simplified slide handling

Let us imagine a histopathology lab with 40 students sitting beside 40 microscopes using 40 slide boxes. During a pathology course a student has to learn about at least a 100 different diseases because they have a characteristic appearance under microscope and have their clinico-pathological importance, or relevance of the alteration to understand basic pathological mechanisms. 40 students sitting beside 40 microscopes using 40 slide boxes each with 100 slides gives 4000 slides to be taken care of whenever the stain faints, a slide brakes or just mysteriously disappears.

### Standardisation

Reproducing 100 slides showing just the same alteration is literally impossible. Serial sectioning represents many different levels of the paraffin embedded samples, and the collection of 100 samples (and additional serial) of rare diseases are very problematic too. Using digital slides only one perfect sample is required. After scanning the digital slide never breaks, the stain does not faint and it shows exactly the same picture to every user.

### Demostration of auxiliary techniques

It is highly expensive to prepare immunostained slides for every student or to show *fluorescence in situ hybridization *(FISH)-slides or other fluorescent material (this requires to equip your lab with 40 fluorescent microscopes), not to mention the continuous collecting of fluorescent samples that are fainting fast. It is not impossible as long one have all the credit, but it would not be worth at all. Scanning IHC-slides or FISH with a fluorescent camera is basically the same process as the simple scanning of H&E slides and provides the above mentioned benefits.

### Unique attributes of digital slides

#### Easier orientation on slides

Every viewer software has a thumbnail view of the slide in the main user interface. For a student who has just passed the anatomy examinations, it is a great help to orient the sample. For example in the case of a gastrointestinal tumour to judge the existence of peritoneal spreading first one has to know which is the mucosal and which is the serosal surface of the gut what could be very difficult for a student especially when the tumour destructs the normal structures.

#### Parallel visualization of slides

With a viewer software it is possible to open multiple slides in parallel. It could be the same slide opened twice and positioned to different areas at different magnifications. For example to show Antony-A and Antony-B areas of a schwannoma. Or open a H&E slide and the IHC slide from the same block to show the immunprofile of a tumour.

#### Placing annotations on digital slides

This feature helps to be tutors rather than teachers, and to apply different teaching-learning methods (e.g.: problem based learning, inquiry-based learning, distance learning, e-learnig etc) with all the supporting information about the sample and the representative areas as annotations or with direct links to scientific databases.

#### Teleconsultation settings

On special slide servers digital slides could be stored and opened from remote locations. Connected to the servers multiple users could open the same slide and create a teleconsultation group where synchronized view is possible as well. With these servers students are able to review the slides before or after practices or preparing for examinations [14-16].

## Conclusions

We are confident, and have showed as well, that digital slides have got numerous advantages over optical slides and are more suitable in education. One of their most important qualities is the potential for standardisation, as they provide the opportunity to show exactly the same material for each student. Access for any digital material, be it a medical database or a digital slide server is far more easier comparing it to the way how students used to loan text books from libraries or consulting on glass slides. The growing importance of informatics in health care and the expected capability of medical doctors to handle many sources of digital materials raise further questions. Medical training programs must add informatics to their curriculum and training materials need to be developed [17]. In special fields of postgraduate training, such as pathology and radiology it is worth considering to organize short courses on digital imaging and photography [18].

## Competing interests

Béla Molnár is the owner of 3DHISTECH Ltd. Budapest. László Fónyad has commercial relationship with 3DHISTECH Ltd. as member of the advisory board for software development, he is no shareholder of the company. László Gerely is an employee of 3DHISTECH Ltd.

## Authors' contributions

Professor András Matolcsy, as director of the 1st Department of Pathology and Experimental Cancer Research, Semmelweis University is responsible for every new installation in the Institute and along with László Fónyad collected the slides that have been scanned for education. László Gerely was responsible for the software development and installation. Mária Cserneki, the IT-administrator of the institute is handling the internal servers and computers of the classroom. László Fónyad and Béla Molnár designed the questionnaires and evaluated the results. All authors read and approved the final manuscript.
